# Multifactorial inhibition of *Candida albicans* by combinations of lactobacilli and probiotic *Saccharomyces cerevisiae* CNCM I-3856

**DOI:** 10.1038/s41598-024-59869-9

**Published:** 2024-04-23

**Authors:** Irina Spacova, Camille Nina Allonsius, Ilke De Boeck, Eline Oerlemans, Ines Tuyaerts, Nele Van de Vliet, Marianne F. L. van den Broek, Luciana Jimenez, Mickaël Boyer, Bertrand Rodriguez, Nathalie Ballet, Sarah Lebeer

**Affiliations:** 1 Laboratory of Applied Microbiology and Biotechnology, Department of Bioscience Engineering, University of Antwerp, Groenenborgerlaan 171, 2020 Antwerp, Belgium; 2 Lesaffre International, Lesaffre Group, Rue Gabriel Péri 137, 59700 Marcq-en-Baroeul, France; 3 Gnosis by Lesaffre, Lesaffre Group, Rue Gabriel Péri 137, 59700 Marcq-en-Baroeul, France

**Keywords:** *Candida*, Yeast, *Lactobacillus*, Antimicrobial, Anti-hyphae, Antifungal agents, Applied microbiology, Biotechnology

## Abstract

Strategies against the opportunistic fungal pathogen* Candida albicans* based on probiotic microorganisms represent a promising alternative to traditional antifungals. Here, we investigated the effects of *Lactobacillaceae* isolates from fermented foods or the human vagina, alone or in combination with the probiotic yeast *Saccharomyces cerevisiae* CNCM I-3856, against *C. albicans *in vitro. Nine out of nineteen tested strains of *Lactobacillaceae* inhibited growth of *C. albicans* with inhibition zones of 1–3 mm in spot assays. Five out of nineteen lactobacilli tested as such or in combination with *S. cerevisiae* CNCM I-3856 also significantly inhibited *C. albicans* hyphae formation, including *Limosilactobacillus fermentum* LS4 and *L. fermentum* LS5 resulting in respectively 62% and 78% hyphae inhibition compared to the control. Thirteen of the tested nineteen lactobacilli aggregated with the yeast form of *C. albicans*, with *Lactiplantibacillus carotarum* AMBF275 showing the strongest aggregation. The aggregation was enhanced when lactobacilli were combined with *S. cerevisiae* CNCM I-3856. No significant antagonistic effects were observed between the tested lactobacilli and S. cerevisiae CNCM I-3856. The multifactorial activity of *Lactobacillaceae* strains alone or combined with the probiotic *S. cerevisiae* CNCM I-3856 against *C. albicans* without antagonistic effects between the beneficial strains, paves the way for developing consortium probiotics for in vivo applications.

## Introduction

In healthy individuals, *Candida albicans* is a common commensal of the skin, the oropharynx, the gastrointestinal and the vaginal tract^1^. The fungus can however shift to an opportunistic pathogen and cause infections, ranging from superficial infections of the mucosa to invasive, life-threatening disease. The most prevalent *C. albicans* infection is vulvovaginal candidosis (VVC), which affects approximately 75% women at least once during their lives, with reported risk factors including diabetes and being part of a couple^2,3^. Other *Candida* infections, including oral and oropharyngeal candidosis, are common in immunocompromised individuals^1,4,5^. Standard treatment of *C. albicans* infections consists of azole therapy, however the development of azole resistance in *Candida* species is rising and worrisome^6^. Furthermore, these standard treatments can also cause side effects, including skin irritation, redness, gastrointestinal disorders and hepatotoxicity^7^. In addition, treated *Candida* infections are often followed by relapses; for instance, 103–172 million women annually suffer from recurrent VVC after an initial infection, with more than four confirmed VVC episodes per year^8^. This is possibly due to aggravated microbiome dysbiosis by azole use, and we recently observed a trend of reduced endogenous lactobacilli after fluconazole treatment^9^. Altogether, this signifies the need for novel therapeutic strategies.

An increasing number of clinical studies show the safety and efficacy of probiotic microorganisms to prevent or treat VVC^10^. Different study designs have been explored, with different follow-up durations, different routes of probiotic administration, or treatment combinations (the probiotic alone, a mixture of probiotics or in an adjuvant setting with azole treatment), mostly with a focus on oral intake. Several studies show that oral (as a food supplement) and vaginal (generally as a drug, with a more complex regulatory route) intake of different probiotic species and strains is associated with improved clinical signs^11–13^. In addition, no side effects have been observed or reported upon probiotic use in healthy women^14^ or women suffering from VVC^11,15,16^.

Despite the promising results from several trials with specific probiotic microorganisms, their multifactorial biological activity is often not fully understood. Yet, substantiation of the biological activity underlying probiotic efficacy can refine strain selection, improve combinations of different microbial strains based on complementary or synergistic properties, allow for patient stratification between responders and non-responders and facilitate regulatory approval. Several studies have evaluated possible mode of actions of individual *Saccharomyces* and *Lactobacillaceae* strains such as *Lactobacillus gasseri* JCM 1131, *Lactobacillus crispatus* JCM 118*5, Lacticaseibacillus rhamnosus* GR-1 and *Limosilactobacillus reuteri* RC-14 in vitro. These include the production of antimicrobial molecules and their direct growth-inhibitory and biofilm-inhibitory activity^17,18^, anti-adhesive capacity through co-aggregation or site exclusion^19,20^, and interference in hyphae formation, which is an important step in the infectious process and key for the ability of *C. albicans* to invade mucosal epithelial cells^9,21,22^. We have recently discovered that lactobacilli occur in modules or guilds of interacting bacteria in the vagina, with *Lactobacillus crispatus, Lactobacillus jensenii* and *Limosilactobacillu*s taxa co-occurring^23^. However, a functional role for these modules, the interacting taxa, with *Limosilactobacillus* taxa in particular, is currently underexplored. Similarly, while probiotic strains are routinely combined during clinical evaluation against VVC^24^, their interacting effects (such as lack of antagonism and possibility for synergism) are often not explored during the in vitro evaluation of mode-of-actions.

In this study, we aimed to explore combinations of lactobacilli (in particular *Lim. fermentum*) and the yeast *Saccharomyces cerevisiae* CNCM I-3856 to target *Candida* infections in the vagina and other mucosal surfaces such as the oral cavity. *S. cerevisiae* CNCM I-3856 was previously reported to be effective against *Candida* infections in vitro and in animals^25,26^. *S. cerevisiae* CNCM I-3856 was also shown to be able to migrate from the gut to the vagina^27^, making a oral food supplementation feasible to target the vagina. However, this yeast does not always result in long-term colonization of the vagina in healthy subjects after oral administration^27^. In addition, *S. cerevisiae* is not a dominant member of the vaginal microbiota^9,16^. Therefore, here we investigated the multifactorial biological activity of probiotic *S. cerevisiae* CNCM I-3856 in combination with *Lactobacillaceae* strains from vaginal and food origin against *C. albicans*. We tested three vaginal *Lactobacillaceae* species, namely *Lactobacillus crispatus, Lactobacillus johnsonii* and *Lacticaseibacillus rhamnosus*, which persist naturally or upon administration in the vagina, at least temporarily^9,16,23^. In addition, we tested multiple *Lim. fermentum* strains isolated from food sources suitable for oral application. Sourdough is characterized by a microbial ecosystem comprised of lactic acid bacteria and yeasts that undergo beneficial metabolic interactions with each other^28^. Thus, sourdough lactobacilli isolates were included because of less risk of undergoing antagonistic interactions with *S. cerevisiae* CNCM I-3856. Another important reason to include lactobacilli isolates from sourdough and vegetable fermentations was that these genera and species of lactobacilli are naturally found in the human gastrointestinal tract^29,30^, while we have also recently demonstrated that several of these taxa can also be routinely isolated from the human vagina^23^. We evaluated potential synergistic and absence of antagonistic effects between the yeast and the selected bacterial species for the development of probiotic combinations of the well-studied *S. cerevisiae* CNCM I-3856 with at least one *Lactobacillaceae* strain, and to better understand the interactions of the probiotic *S. cerevisiae* CNCM I-3856 with lactobacilli potentially found in the gastrointestinal and vaginal microbiome.

## Material and methods

### Microbial strains used in this study

The clinical isolate *C. albicans* SC5314^31^ was used as a pathogenic strain. The probiotic yeast *S. cerevisiae* CNCM I-3856^15,25–27^ was obtained from a commercially available probiotic product (Gnosis by Lesaffre, Marcq-en-Baroeul, France) and used either as overnight culture, cell-free culture supernatant or commercial dried powder^26^. A total of 19 *Lactobacillaceae* strains were selected as explained above in the “Introduction” section, and used for the analyses as detailed in Table 1 either as overnight culture or cell-free culture supernatant. We used live lactobacilli and not a powder formulation, as this is how they are found in the gastrointestinal tract and the vagina. Yeasts were grown in yeast extract peptone dextrose (YPD) broth (Carl Roth), while lactobacilli were grown in De Man, Rogosa and Sharpe (MRS) broth (Difco). Growth of microorganisms was measured using spectrophotometer at 600 nm.

**Table 1:** Lactobacilli isolates used in this study and their origin.

Strain	Full strain name	Origin	Reference/source
AMBV012	*Lactobacillus crispatus* AMBV012	Human vagina	^32,33^
AMBV023	*Lactobacillus johnsonii* AMBV023	Human vagina	^32,33^
AMBV083	*Lacticaseibacillus rhamnosus* AMBV083	Human vagina	^32,33^
AMBF275	*Lactiplantibacillus carotarum* AMBF275	Fermented plants and vegetables	^34^
AMBF471	*Limosilactobacillus reuteri* AMBF471	Fermented plants and vegetables	^35^
LS1	*Lactiplantibacillus plantarum* LS1	Sourdough sourced from Hungary	Lesaffre International
LS2	*Lactiplantibacillus plantarum* LS2	Sourdough sourced from Russia	Lesaffre International
LS3	*Lactiplantibacillus plantarum* LS3	Sourdough sourced from Poland	Lesaffre International
LS4	*Limosilactobacillus fermentum* LS4	Sourdough sourced from the Czech Republic	Lesaffre International
LS5	*Limosilactobacillus fermentum* LS5	Sourdough sourced from Serbia and Montenegro	Lesaffre International
LS6	*Lacticaseibacillus paracasei* LS6	Sourdough sourced from China	Lesaffre International
LS7	*Lactiplantibacillus plantarum* LS7	Sourdough sourced from Russia	Lesaffre International
LS8	*Lactiplantibacillus plantarum* LS8	Sourdough sourced from Poland	Lesaffre International
LS9	*Lactiplantibacillus plantarum* LS9	Sourdough sourced from China	Lesaffre International
LS10	*Lactiplantibacillus plantarum* LS10	Sourdough sourced from China	Lesaffre International
LS11	*Lacticaseibacillus paracasei* LS11	Sourdough sourced from the Czech Republic	Lesaffre International
LS12	*Lacticaseibacillus paracasei* LS12	Sourdough sourced from Hungary	Lesaffre International
LS13	*Lacticaseibacillus paracasei* LS13	Sourdough sourced from Poland	Lesaffre International
LS14	*Lacticaseibacillus paracasei* LS14	Sourdough sourced from Poland	Lesaffre International

### Spot assay for monitoring growth inhibition of *C. albicans*

Spot assay against *C. albicans* was performed as previously described^9^ with minor modifications. Briefly, 2 µL of overnight cultures of lactobacilli or *S. cerevisiae* CNCM I-3856 were spotted on solid agar at set distances from each other (1 cm, 1.5 cm or 2 cm, allowing to evaluate individual and possible synergistic effects). After 24 h of spots incubation, an overlay of a soft YPD agar (0.5% agar) (Carl Roth) containing *C. albicans* SC5314 at 4 × 10^7^ CFU/mL was poured over the spots. After overnight incubation at 37 °C in aerobic conditions, growth of *C. albicans* SC5314 was evaluated, and inhibition zones were measured in mm.

### Time course analysis of *C. albicans* growth inhibition and of *S. cerevisiae* and lactobacilli growth

Time course analysis of *C. albicans* growth inhibition was performed as previously described^19^ with minor modifications. Briefly, spent culture supernatants of overnight *Lactobacillaceae* and *S. cerevisiae* CNCM I-3856 cultures was obtained by centrifugation (10 min, 2000 g) and filtration with a 0.22 μm sterile syringe filter (VWR). The following supernatants from overnight cultures were tested against *C. albicans* SC5314: (1) supernatant of *Lactobacillaceae* as such in MRS broth; (2) supernatant of *S. cerevisiae* as such in YPD broth; (3) mixture of *Lactobacillaceae* and *S. cerevisiae* CNCM I-3856 supernatants in MRS and YPD, respectively. The supernatant was supplemented to fresh growth medium of *C. albicans* in a 1:10 ratio for a total volume of 200 µL and added to a flat-bottom 96-well plate (VWR). Each well was inoculated with an overnight culture of *C. albicans* SC5314 at 1% (v/v). Conditions with *C. albicans* as such, *C. albicans* with MRS at pH 4 and *C. albicans* with miconazole (80 µg/mL) were included as control conditions. Growth of *C. albicans* in continuous shaking conditions and 37 °C was evaluated over 24 h by measuring the optical density at 600 nm every 30 min in each well of the 96-well plate with the Synergy HTX multimode reader.

In addition, the same methodology was used to test the effects of vaginal *Lactobacillaceae* supernatants on the growth of *S. cerevisiae* CNCM I-3856, and the effects of the *S. cerevisiae* CNCM I-3856 supernatant on the growth of vaginal *Lactobacillaceae* strains.

### *C. albicans* co-aggregation/agglutination and binding assay with *S. cerevisiae* and lactobacilli

#### Co-aggregation/agglutination with *C. albicans* yeast cells

The non-fluorescent co-aggregation assay was based on^26^ with several adjustments. Overnight *C. albicans* SC5314 cultures were washed twice (1500 × g, 10 min) and resuspended in phosphate buffered saline (PBS) to obtain a 1% (w/v) cell suspension. *S. cerevisiae* CNCM I-3856 from commercial dried powder was also resuspended in PBS to obtain a 1% w/v cell suspension. *Lactobacillaceae* cultured overnight were adjusted to a concentration of 10^10^ CFU/mL. After vortexing, 50 µL of *C. albicans* suspension, 50 µL of *S. cerevisiae* suspension and 25 µL of lactobacilli were added to the wells of a U-bottom 96-well plate (VWR). All *Lactobacillaceae* and *S. cerevisiae* were also tested as such with *C. albicans*. The plate was incubated at room temperature with gentle shaking. After 10 min and 1 h, the co-aggregation rate was microscopically evaluated using the Olympus CX41 light microscope and Olympus U-CMAD3 camera. Scores from 0 to 4 were given for each condition, as described by^26^, with a score of 0: no aggregation; 1: aggregates with small clusters; 2: aggregates with larger numbers of yeasts; 3: clumps visible with the naked eye containing large numbers of yeast cells; 4: maximum score for large clumps visible with the naked eye in the well center. For conditions where even higher aggregation was observed, we rationalized that a higher score was needed and indicated this with 4+.

#### Co-aggregation/agglutination with *C. albicans* after hyphae induction

Furthermore, fluorescent labelling of *C. albicans* SC5314 and *S. cerevisiae* CNCM I-3856 partially based on Pericolini et al.^26^ was performed to assess their binding/co-aggregation. Overnight culture pellet containing 1 × 10^7^ CFU of *C. albicans* was resuspended in 250 µL CalcoFluor White (CFW) (VWR) and stained with 0.5 mg CFW/mL. *S. cerevisiae* CNCM I-3856 at containing 1 × 10^7^ CFU from commercial dried powder was resuspended in 250 µL Fluorescein isothiocyanate (FITC) solution and stained with FITC (F7250-1G, Sigma) at a concentration of 0.4 mg/mL. Labelling was performed at room temperature for 1 h at 250 rpm. After centrifugation for 10 min at 4000 g, the pellet was washed using PBS and resuspended in 1 mL to obtain a concentration of 4 × 10^7^–10^8^ CFU/mL for *C. albicans* and *S. cerevisiae* CNCM I-3856. To assess potential binding based on fluorescence in mixtures of *C. albicans* with *S. cerevisiae* CNCM I-3856, mixtures were made by adding 12.5 µL of the *C. albicans* suspension to 12.5 µL of fetal calf serum (FCS) (Gibco) and 100 µL YPD broth in a 96 U-bottom well plate. After 3 h of incubation under non-shaking conditions at 37 °C 50 µL of the FITC-stained *S. cerevisiae* CNCM I-3856 suspension was added to the wells with *C. albicans* and incubated at room temperature with gentle shaking (200 rpm). After 10 min or 1 h of incubation, 2 µL of the mixtures was used for microscopic evaluation by checking binding between cells or cells and hyphae, and cluster formation between *C. albicans* SC5314 (blue) and *S. cerevisiae* CNCM I-3856 (green). Fluorescence microscopy images were recorded with the Leica DMi8 fluorescence microscope.

### Inhibition of *C. albicans* hyphae formation by *S. cerevisiae* and lactobacilli

Two protocols were implemented to assess inhibition of *C. albicans* SC5314 hyphae formation by *S. cerevisiae* CNCM I-3856 cell-free culture supernatant and *Lactobacillaceae* or their mixtures based on previously developed protocols by Allonsius et al.^19,21^. In the first protocol not implementing fluorescent labeling, mixtures of 10^6^ CFU/mL of *C. albicans* SC5314 with cell-free culture supernatant of *S. cerevisiae* CNCM I-3856, with *Lactobacillaceae* or both were made by adding 50 µL of suspension of each tested microorganism to 125 µL of FCS, supplemented with YPD broth to a total volume of 500 µL. For each condition three technical repeats were included. After 3 h of incubation in non-shaking conditions at 37 °C, 2 µL of the mixtures was used for microscopic evaluation by counting the number of yeast cells and the number of cells forming hyphae. At least 100 cells were counted and ratios of hyphae to yeast cells were calculated and normalized to *C. albicans* as such with FCS. For normalization, the average ratio of hyphae to yeast cells counted for *C. albicans* as such (after incubation with FCS, “*C. albicans* control”) was first calculated. Afterwards, the number of yeast cells and number of hyphae were counted for each repetition of each conditions and the ratios were calculated. These ratios were then divided by the average ratio of hyphae to yeast cells counted for *C. albicans* as such (after incubation with FCS, “*C. albicans* control”).

The second protocol implemented fluorescent labeling to test the effects of live *S. cerevisiae* CNCM I-3856 from commercial dried powder and *Lactobacillaceae* or their mixtures for inhibition of *C. albicans* SC5314 hyphae formation. The two best performing strains from the first hyphal inhibition experiment, namely *L. fermentum* LS4 and LS5, were selected for this experiment to be used as such or in combination with live *S. cerevisiae* CNCM I-3856. *S. cerevisiae* CNCM I-3856 powder was reconstituted in YPD broth after weighing the dried powder 0.1% w/v, and the resuspended powder was shaken for 15 to 20 min at 100 rpm. The concentrations of overnight cultures of *Lactobacillaceae* and yeast were subsequently adjusted to 10^9^ CFU/mL and 10^7^ CFU/mL, respectively. Fluorescent staining of *C. albicans* SC5314 and *S. cerevisiae* CNCM I-3856 was performed as described above for the co-aggregation assay. Inhibition of *C. albicans* hyphae formation by *S. cerevisiae* CNCM I-3856 as commercial powder, overnight culture or supernatant was tested. To assess hyphae formation in mixtures of *C. albicans* with *S. cerevisiae* and/or lactobacilli *L. fermentum* LS4 or *L. fermentum* LS5, mixtures were made by adding 50 µL of suspension of each tested microorganism to 125 µL of FCS, supplemented with YPD broth until 500 µL. For each condition three or four technical repeats were included in a 24 well plate. After 3 h of incubation at 37 °C to allow *C. albicans* hyphae formation, 2 µL of the mixtures was used for microscopic evaluation by counting the number of yeast cells and the number of cells forming hyphae. At least 100 cells were counted and ratios of hyphae to yeast cells were calculated and normalized to *C. albicans* as such with FCS. Fluorescence microscopy images were recorded with the Leica DMi8 fluorescence microscope.

### Statistical analysis

Significant differences between all tested conditions were evaluated in GraphPad Prism version 9.2.0 for Windows (GraphPad Software, San Diego, California USA, www.graphpad.com). Statistical testing was performed using one-way ANOVA (when testing one experimental factor, for example effects of lactobacilli strains) or two-way ANOVA (when testing several experimental factors, for example effects of lactobacilli as such or with *S. cerevisiae*) with Dunnett's multiple comparisons test to identify pairs of conditions with significant differences compared to the control group; p-values < 0.05 were considered significant. Experimental conditions were tested at least in triplicates.

## Results

### Specific strains of lactobacilli as such or in combination with *S. cerevisiae* CNCM I-3856 inhibit growth of *Candida albicans*

A total of 19 lactobacilli isolates of food (sourdough, and fermented plants and vegetables) and vaginal origin were selected for testing against *C. albicans* SC5314 (Table 1).

**Figure 1:**
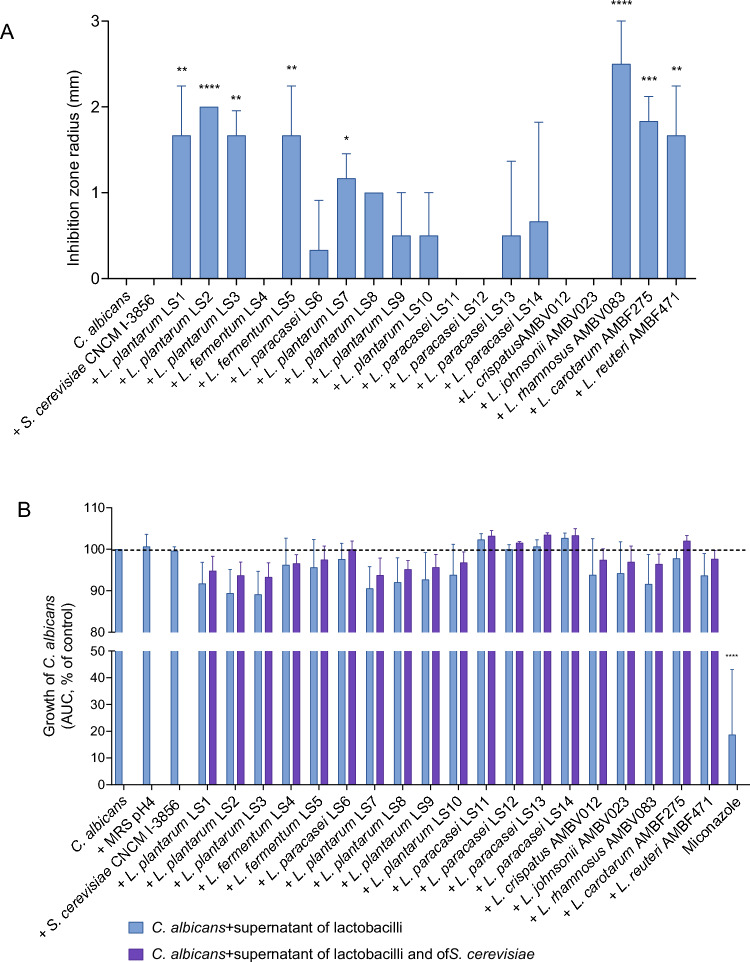
(**A**) Growth inhibition zones in the spot assay against *C. albicans* SC5314 measuring growth inhibition of *C. albicans* by cultures (spots) of live *S. cerevisiae* CNCM I-3856 and the tested lactobacilli as such. (**B**) Growth inhibition of *C. albicans* by supernatant of *S. cerevisiae*, the lactobacilli as such, or the mixture of supernatant of the lactobacilli and supernatant of *S. cerevisiae*. MRS medium at pH 4 was used as an acidic control condition, while miconazole was used as antifungal control. Bars represent the calculated area under the *C. albicans* growth curves and data are expressed as mean ± standard deviation (SD). Significant differences compared to *C. albicans* control (dotted line) are shown (*p < 0.05, **p < 0.001, ***p < 0.001, ****p < 0.0001).

First, we assessed whether live metabolically active lactobacilli or *S. cerevisiae* CNCM I-3856 could inhibit the growth of *C. albicans* by performing spot assays. Almost half of the lactobacilli tested (9/19; 47%) showed growth inhibitory activity against *C. albicans*, with inhibition zones ranging between 1 and 3 mm (Fig. 1A). The largest inhibition zone of 3 mm was observed for *L. rhamnosus* AMBV083. Also *L. plantarum* LS2, *L. fermentum* LS5, *L. paracasei* LS14 and *L. carotarum* AMBF275 showed a strong inhibitory capacity against *C. albicans* with approximately 2 mm inhibition zones. However, no specific growth inhibition of *C. albicans* by *S. cerevisiae* CNCM I-3856 was detected.

Subsequently, we evaluated whether secreted metabolites of lactobacilli, secreted metabolites of *S. cerevisiae* or their combination inhibited the growth of *C. albicans* in a time-course assay*.* Cell-free culture supernatants of 11 out of 19 tested lactobacilli inhibited the growth of *C. albicans* by at least 5% (Fig. 1B). This was not the case for MRS at pH 4, which was used as a control, suggesting an additional effect of lactobacilli metabolites in addition to the acidic environment. The combinations of lactobacilli with the supernatant of *S. cerevisiae* CNCM I-3856 led to similar *C. albicans* growth inhibition, without significant differences compared to the supernatants of lactobacilli as such. Supernatant of *S. cerevisiae* alone did not inhibit *C. albicans* growth under the tested conditions (Fig. 1B). Importantly, the supernatant of *S. cerevisiae* CNCM I-3856 did not lead to significant longitudinal inhibition of the growth of the vaginal lactobacilli isolates (Fig. S1B). Similarly, the culture supernatants of the vaginal lactobacilli did not significantly reduce the growth of *S. cerevisiae* CNCM I-3856 (Fig. S1A).

Altogether, *L. plantarum* LS1, *L. plantarum* LS2, *L. plantarum* LS3, *L. plantarum* LS7 and *L. rhamnosus* AMBV083 showed the strongest antimicrobial effects on *C. albicans* in both assays performed here. The longitudinal effects of these best performing lactobacilli strains’ supernatants on the growth curves of *C. albicans* are shown in the Supplementary Material (Fig. S2).

### *S. cerevisiae* CNCM I-3856 as such and in combination with lactobacilli promotes *Candida albicans* aggregation

Another possible biological mechanism of *S. cerevisiae* and lactobacilli to prevent *Candida* adhesion and subsequent infection is by aggregating with *C. albicans,* because aggregation promotes closer contact with potential antimicrobial metabolites and can block adhesion to target host sites by *C. albicans*. Here, we assessed the cluster formation of *C. albicans* with lactobacilli, with *S. cerevisiae* or in combination after short-term co-incubation (10 min) by microscopic evaluation.

**Figure 2:**
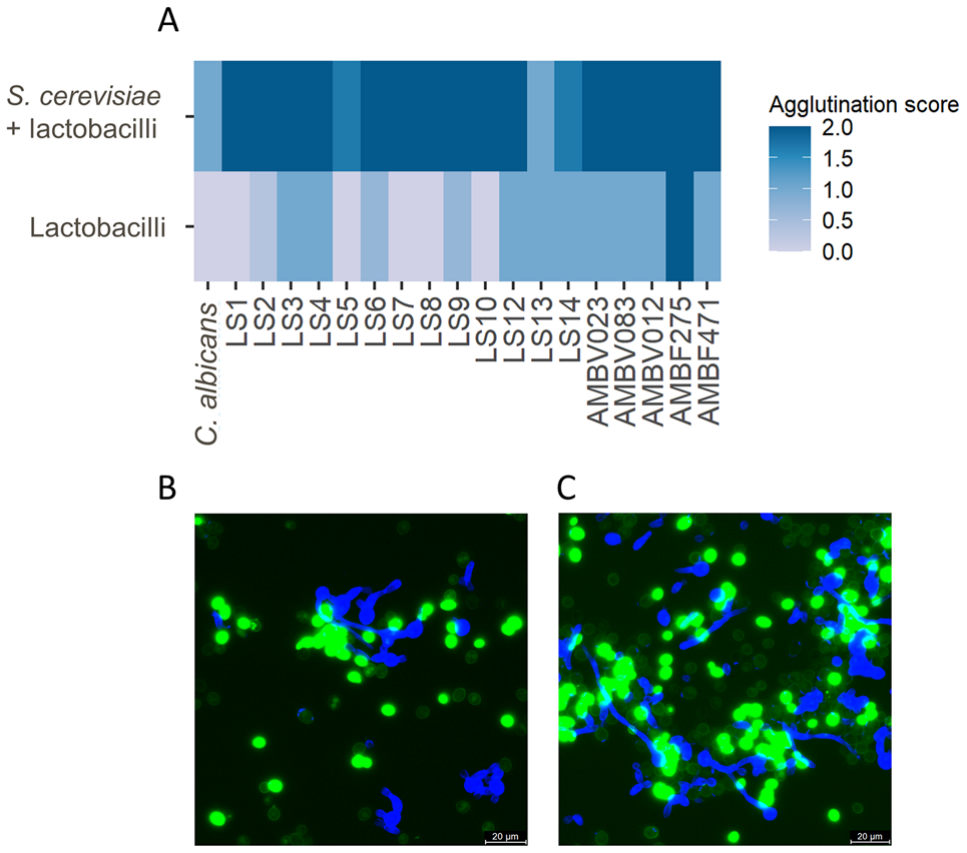
(**A**) Aggregation scores of the yeast form of *C. albicans* SC5314 cells with lactobacilli as such, or in co-culture with dried *S. cerevisiae* CNCM I-3856 after 10 min of incubation; (**B**, **C**) aggregation of *S. cerevisiae* CNCM I-3856 (in green, stained with FITC) with *C. albicans* SC5314 after hyphae induction (in blue, stained with CFW) after 10 min (**B**) or 1 h (**C**) of co-incubation. The clusters formed were scored from 0 (no clusters) to 4+ based on their size according to^26^.

*S. cerevisiae* aggregated with the yeast form of *C. albicans* after 10 min of incubation (Fig. 2A; Supplementary Table S1). The majority (13/19; 68%) of the lactobacilli also aggregated with the yeast form of *C. albicans* cells after 10 min of incubation with a score of 1 out of 4: *L. plantarum* LS3, *L. fermentum* LS4, *L. paracasei* LS12, *L. paracasei* LS13, *L. paracasei* LS14, *L. crispatus* AMBV012, *L. johnsonii* AMBV023, *L. rhamnosus* AMBV083 and *L. reuteri* AMBF471. *L. carotarum* AMBF275 showed the strongest aggregation with *C. albicans* with a score of 2 out of 4.

Remarkably, the combination of *S. cerevisiae* and lactobacilli gave a strong aggregating effect after 10 min of incubation, which was observed for all lactobacilli. While the aggregating effect was clear from microscopic evaluation also after a longer incubation of 1 h (Supplementary Fig. S3), the synergistic effect of *S. cerevisiae* and lactobacilli on the yeast form of *C. albicans* aggregation was not strengthened by the longer incubation (Supplementary Table S2).

Additionally, a visual indicative assessment of *S. cerevisiae* CNCM I-3856 binding to *Candida albicans* after hyphae induction was performed using fluorescently stained *S. cerevisiae* CNCM I-3856 and *Candida albicans*. Aggregation of the hyphal form of *C. albicans* with *S. cerevisiae* CNCM I-3856 was visually confirmed to occur via fluorescent microscopy with differentially stained cells (Fig. 2B) after both 10 min and 1 h of co-incubation.

### Lactobacilli as such or in combination with *S. cerevisiae* CNCM I-3856 inhibit hyphae formation by *Candida albicans*

**Figure 3:**
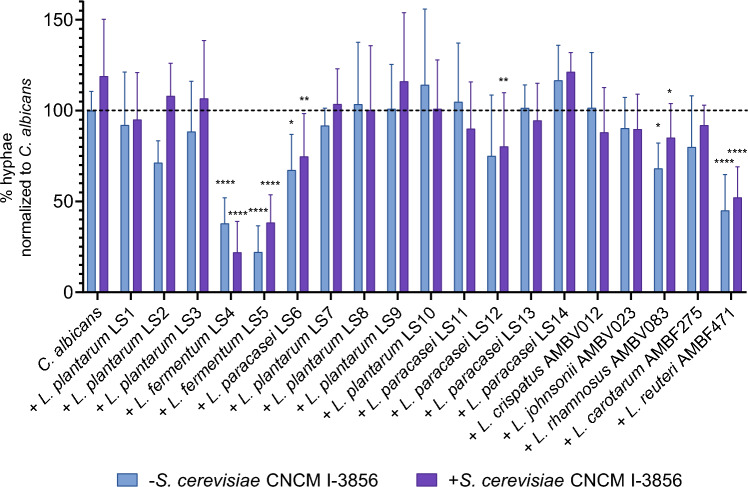
*C. albicans* SC5314 hyphal formation inhibition during co-incubation with lactobacilli as such or combined with cell-free culture supernatant of *S. cerevisiae* CNCM I-3856. A concentration of 1/4 FCS was used to induce hyphae formation. Results are represented as ratio against a total number of 100 yeast cells and normalized against *C. albicans* as such. Data are expressed as mean ± SDs. Significant differences compared to *C. albicans* control are shown with an asterisk (* p < 0.05, ** p < 0.001, **** p < 0.0001).

Lastly, we tested the effects of *S. cerevisiae* and lactobacilli on hyphae formation of *C. albicans* necessary for this pathogen to invade mucosal epithelial cells^9,19,22^. In the first set of experiments, the effects of live lactobacilli as such and in combination with cell-free culture supernatant of *S. cerevisiae* were tested for their ability to inhibit hyphae formation by *C. albicans* (Fig. 3 and Supplementary Fig. S4)*.* Significant inhibition of hyphae formation was demonstrated for *L. fermentum* LS4 (by 62 ± 14% compared to 100% *Candida* control), *L. fermentum* LS5 (by 78 ± 14%), *L. paracasei* LS6 (by 33 ± 20%), *L. johnsonii* AMBV083 (by 32 ± 14%) and *L. reuteri* AMBVF471 (by 61 ± 24%) as such. This was also the case for combinations with supernatant of *S. cerevisiae* CNCM I-3856, especially for *L. fermentum* LS4 (inhibition of hyphae formation by 78 ± 17% compared to 100% *Candida* control).

**Figure 4:**
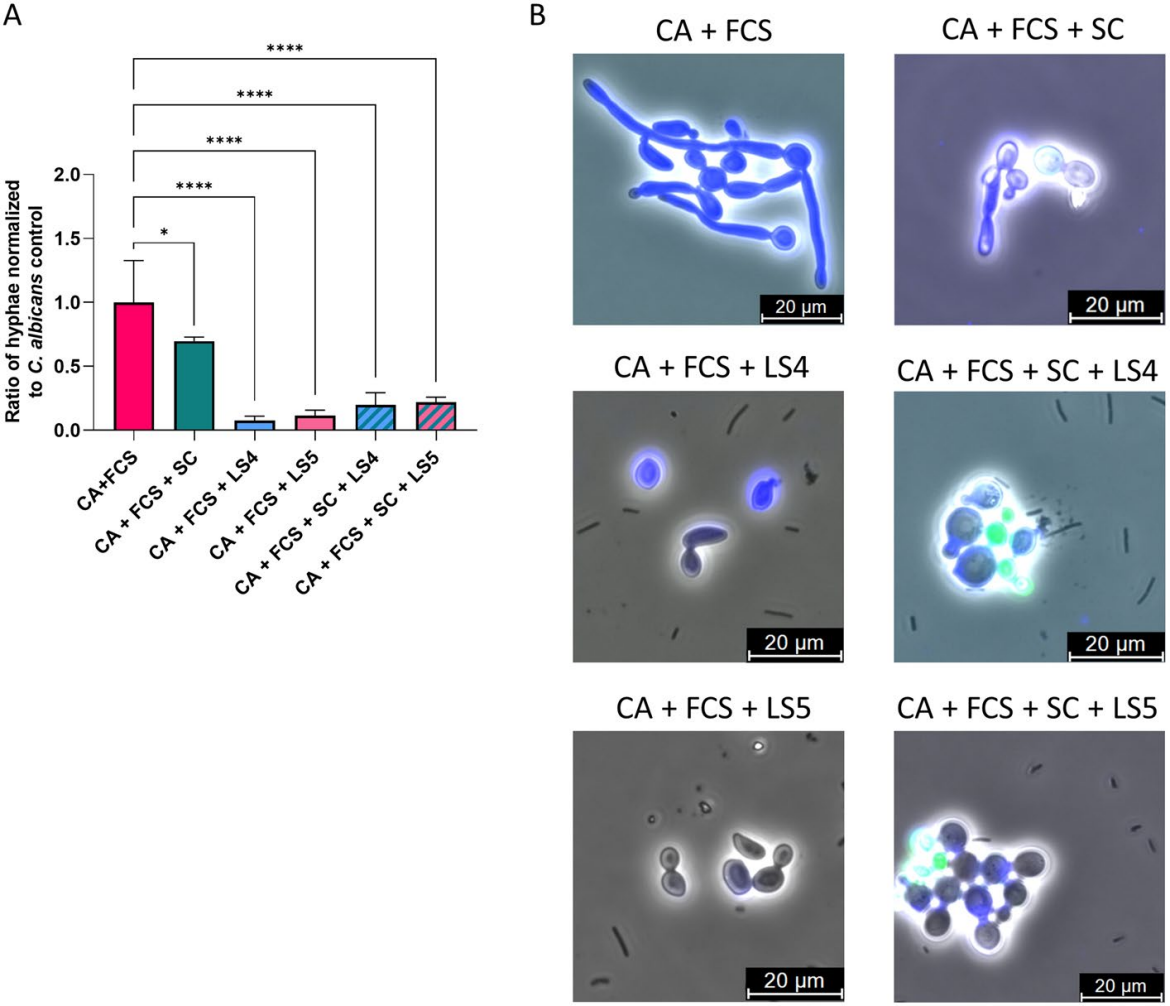
(**A**) Hyphal induction of *C. albicans* SC5314 during co-incubation with live *S. cerevisiae* CNCM I-3856 together with *L. fermentum* LS4 or LS5, or with *L. fermentum* LS4 or LS5 as such. A concentration of 1/4 FCS was used to induce hyphae formation in *C. albicans* (CA + FCS). *S. cerevisiae* CNCM I-3856 powder was used together with *C. albicans* with FCS (CA + FCS + SC), or also with the different lactobacilli (CA + FCS + SC + LS4; CA + FCS + SC + LS5). Also conditions with *C. albicans* and lactobacilli without *S. cerevisiae* were included (CA + FCS + LS4; CA + FCS + LS5). Results are represented as ratio against a total number of 100 yeast cells and normalized against *C. albicans* as such. Data are expressed as mean ± SD. Significant differences compared to *C. albicans* with FCS conditions (CA + FCS) are shown with an asterisk (* p < 0.05, **** p < 0.0001); (**B**) fluorescence microscopy examples from the experiment on hyphal induction of *C. albicans* during co-incubation with live *S. cerevisiae* CNCM I-3856 together with *L. fermentum* LS4 or LS5. A concentration of 1/4 FCS was used to induce hyphae formation in *C. albicans* (CA + FCS), while *C. albicans* (CA) as such had no FSC added. *S. cerevisiae* CNCM I-3856 powder was used together with *C. albicans* with FCS (CA + FCS + SC), or also with the different lactobacilli (CA + FCS + SC + LS4; CA + FCS + SC + LS5; CA + FCS + SC + LS7).

Based on the results of Fig. 3, the two best performing strains from the first hyphal inhibition experiment showing the strongest inhibition of *C. albicans* hyphae formation, namely *L. fermentum* LS4 and LS5, were selected to be further evaluated in a second set of experiments. In these experiments, they were combined with fluorescently labelled live *S. cerevisiae* CNCM I-3856 from dried powder and tested for their ability to inhibit hyphae formation by *C. albicans.* Significant inhibition of *C. albicans* hyphae formation was observed both by *L. fermentum* LS4 (by 92.5 ± 4% compared to 100% *Candida* control) and *L. fermentum* LS5 (by 88.5 ± 4%) as such and their combination with live *S. cerevisiae* CNCM I-3856 (Fig. 4).

## Discussion

**Figure 5:**
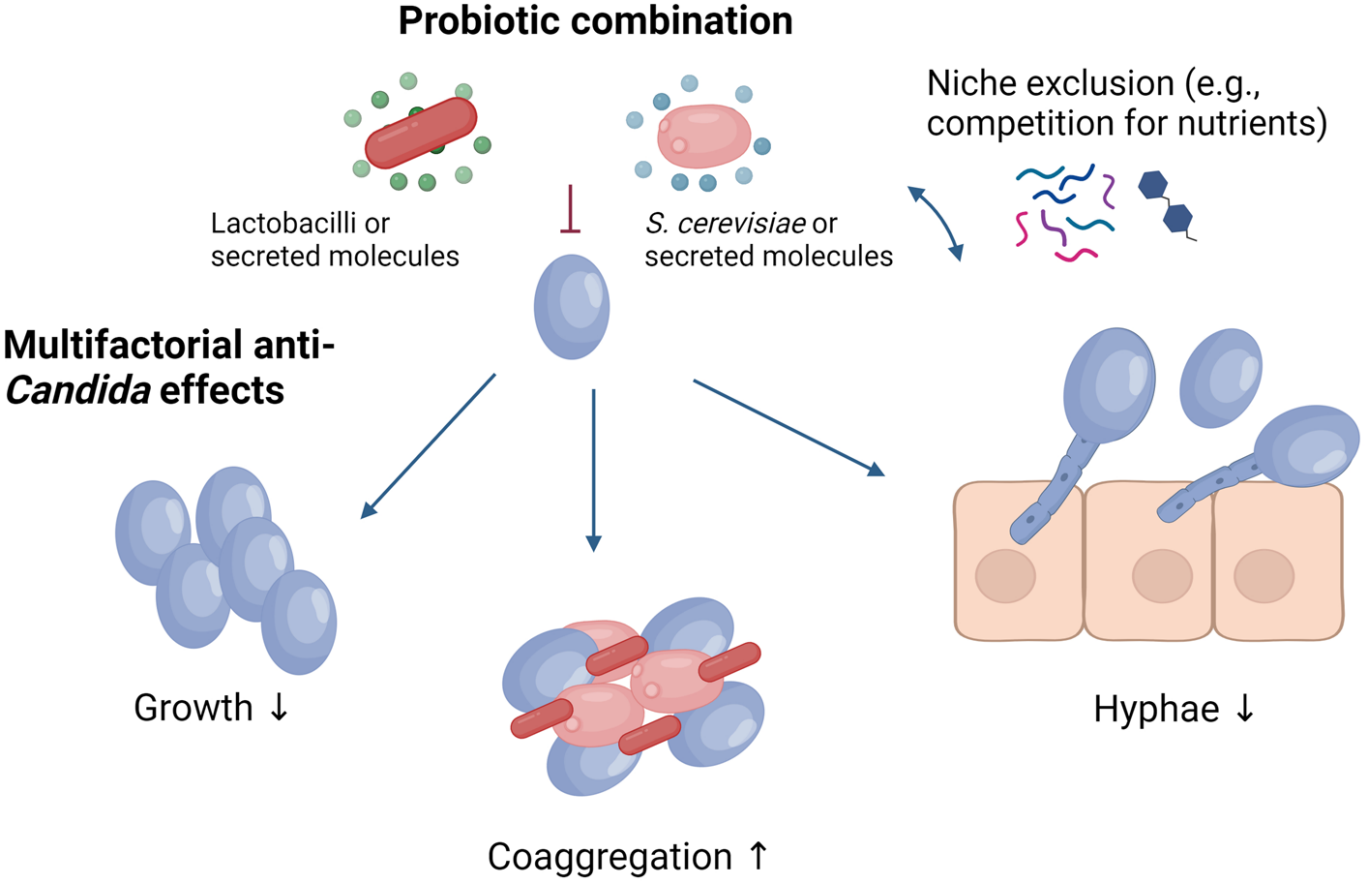
Postulated biological activity through which *S. cerevisiae* CNCM I-3856 and *Lactobacillaceae* strains inhibit *C. albicans*. The contribution of each mechanism can vary depending on the microbial strains used and the in vivo context (*e.g*., the microbiome). Created with BioRender.com.

In this study, we investigated the in vitro efficacy of probiotic *Saccharomyces cerevisiae* CNCM I-3856 and *Lactobacillaceae* strains against *C. albicans* SC5314 through different potential types of biological activity. We showed that lactobacilli alone or in combination with *S. cerevisiae* CNCM I-3856 lead to growth inhibition, agglutination and hyphae inhibition of *C. albicans *in vitro*,* thus acting against at least three key aspects involved in *C. albicans* pathogenesis in vivo (Fig. 5). The significance of the observed lactobacilli-mediated effects was species-specific (e.g., for hyphae inhibition) or even strain-specific (e.g., for *C. albicans* growth inhibition) for each of the discussed types of biological activity summarized in Fig. 5. The tested *L. fermentum* strains most efficiently inhibited hyphae formation, but also other strains showed significant anti-hyphae activity, including *L. paracasei* and *L. reuteri* strains. The tested *L. carotarum* strain scored the highest capacity for *C. albicans* aggregation. *Candida* growth inhibition in the tested conditions was modest, with predominantly *L. plantarum* and *L. rhamnosus* demonstrating the highest inhibition of *C. albicans* growth via secreted metabolites*.* For the species *L. paracasei,* we found that the activity was strongly strain-specific, with strains showing no growth inhibition (LS11, LS12) and strains that did (LS13, LS14).

Synergistic effects between lactobacilli and the yeast *S. cerevisiae* CNCM I-3856 against vaginal *C. albicans* in our work were observed at the level of agglutination in the tested *i*n vitro set-ups. A tendency towards a stronger anti-hyphae effect of *L. fermentum* LS4 combined with *S. cerevisiae* CNCM I-3856 compared to *L. fermentum* LS4 was also observed in some experiments, however the effect *L. fermentum* LS4 and LS5 as such against hyphae was so pronounced that the demonstration of synergy with *S. cerevisiae* CNCM I-3856 was complicated in our in vitro experimental set-ups. Of note, other ecological mechanisms active in vivo and synergistic effects with other microbiome members that can further boost the efficacy of the *S. cerevisiae* CNCM I-3856 and lactobacilli are not excluded. Such synergistic in vivo effects could explain the more promising results against *Candida* hyphae formation observed in a mouse model of VVC upon *S. cerevisiae* CNCM I-3856 administration, in addition to differences in experimental set-ups^26^. Likewise, a previous in vivo clinical study in women demonstrated that orally administered *S. cerevisiae* CNCM I-3856 can serve as an effective adjuvant therapy to conventional VVC treatment through inhibiting vaginal *Candida* proliferation^15^.

Importantly, we did not observe significant antagonistic activity between *S. cerevisiae* CNCM I-3856 and the tested lactobacilli regarding *C. albicans* inhibition, highlighting that they can be applied together without loss of activity. Furthermore, it is plausible that *S. cerevisiae* CNCM I-3856 will not have major antagonistic effects with the lactobacilli comprising the natural vaginal microbiome, as we have shown for the vaginal lactobacilli in this paper, and recently demonstrated in healthy women^14^. This is also relevant for oral *S. cerevisiae* CNCM I-3856 administration, as it has previously been demonstrated that after its oral administration in women *S. cerevisiae* CNCM I-3856 can be recovered from vaginal samples of at least 18% to 21% of participants, depending on the analysis^14,27^. Also in the gut, a combined effect of *S. cerevisiae* CNCM I-3856 and strains of *L. plantarum*, *L. rhamnosus* and *L. fermentum* strains can be hypothesized, as these lactobacilli are naturally present in fermented foods^29^ and, consequently, in the human gut depending on the individual diet^30^.

Our results provide insight in the biological activity underlying the effects observed in vivo in the studies of Pericolini et al.^26^ and Cayzeele-Decherf et al.^15^. The action of lactobacilli and *S. cerevisiae* CNCM I-3856 in vivo is likely multifactorial, as summarized in Fig. 5. While in this study we have not explored which exact molecules of lactobacilli are responsible for *Candida* hyphae or growth inhibition, we have previously already demonstrated that one of the most prominent surface molecules of lactobacilli, exopolysaccharides (EPS), could inhibit growth and hyphae formation^19^, but the activity was even higher for the peptidoglycan hydrolase major secreted protein 1 (Msp1) with chitinase functionality produced by *Lacticaseibacillus* strains^21^. Related to direct antimicrobial interactions, the main secreted molecules in lactobacilli are d- and l-lactic acid, which have been linked to inhibition of *C. albicans* virulence by multiple studies^19,21,36^. In addition to their impact on the different virulence factors of *C. albicans* (growth, adhesion via aggregation and hyphae formation), the lactobacilli and *S. cerevisiae* CNCM I-3856 cells may also interact with each other and with *C. albicans* via other mechanisms, which might become more prominent in vivo. For example, ecological interactions such as nutrient competition and metabolite production were not explored in this study, yet competition for nutrients has been described between *S. cerevisiae* and *Candida*^37^ and thus has been included in potential mechanisms in Fig. 5. Finally, another potential mechanism might be through microbiome modulation. Although our recent exploratory, randomized, double-blind, placebo-controlled clinical study (n = 60) only showed limited effect of probiotic supplementation of *S. cerevisiae* CNCM I-3856 on the fungal and bacterial community of healthy women^14^, the in vitro results of the current manuscript suggest that the effects of *S. cerevisiae* CNCM I-3856 in combination with specific lactobacilli providing optimal probiotic action are promising to further investigate in women suffering from vaginal infections.

Several limitations of our study lie in the fact that specific in vitro assays were implemented, which do not always reflect in vivo conditions. First, the standard deviation per condition was rather high when in vitro results of several experimental repeats were combined, which can be explained by the intrinsic variability of tripartite biological assays combining three microorganisms or their supernatants (*Candida, Saccharomyces* and lactobacilli). Indeed, some experimental groups presented values greater than 100% in the hyphae formation experiment relative to the average of the *Candida* control condition. Another example is that the supernatants of lactobacilli and *S. cerevisiae* used in the anti-*Candida* growth assays had to be diluted, possibly reducing their biological activity. In our study, careful statistical testing was implemented to elucidate whether the observed effects could have occurred by chance. Another limitation of in vitro assays is that dedicated choices have to be made regarding the biological mode of action to focus on, and in future work also the effects of *S. cerevisiae* CNCM I-3856 and lactobacilli against *C. albicans* biofilms should be explored. Second, the probiotic activity is caused not only by individual probiotic species as they are studied in vitro, but also by their multi-microbial interaction with resident microbial communities in vivo, making it important to take whole microbial communities into consideration^38^. To address the study limitations, in the future we suggest to implement the tested microorganisms in innovative assays allowing to better mimic the human body environment (e.g., organoid systems), monitor relevant health read-outs in vitro (e.g. vagina-on-a-chip) and potentially add synthetic microbial communities or whole microbiomes resident in the human body. Furthermore, additional in vivo studies should be conducted with detailed microbiome and metabolomics read-outs to better understand the role of *S. cerevisiae* CNCM I-3856 and lactobacilli against *C. albicans* as part of an integrated system in the female gut and vagina.

Taken together, our results pave the way for different probiotic combinations or consortia, for example opening up possibilities for combining specific *L. plantarum*, *L. rhamnosus, L. fermentum* and *L. carotarum* probiotics with *S. cerevisiae* CNCM I-3856. On the other hand, our findings on the strain-specific effects of lactobacilli in combination with *S. cerevisiae* CNCM I-3856 are especially important considering the knowledge that the vaginal microbiome of women is dominated by different species of lactobacilli. Our recent results from the Isala study in a large Belgian cohort show that *L. plantarum*, *L. rhamnosus* and *L. fermentum* are naturally present in the female vagina at high prevalence, but not high relative abundance^23^, suggesting that *S. cerevisiae* CNCM I-3856 might be more effective in some women due to their natural gut or vaginal microbiome composition. In line with the Isala study, our results further underline the potential keystone function of *Limosilactobacillus* to promote health in the vaginal niche^23^. Considering that the vaginas of healthy women are colonized with different lactobacilli and it is not yet clear how differences in dominant lactobacilli can be explained, personalized therapies with different combination of probiotic lactobacilli and *S. cerevisiae* CNCM I-3856 might be needed for positive clinical outcomes.

## Conclusion

We have demonstrated the potential multifactorial biological activity of specific *Lactobacillaceae* strains alone or combined with the probiotic *S. cerevisiae* CNCM I-3856 against *C. albicans *in vitro, which was more pronounced for anti-hyphae and agglutination action and was less significant for growth inhibition under the tested conditions. Our results show strain-specific anti-*Candida* modes of action of lactobacilli and no significant antagonistic effects between lactobacilli and *S. cerevisiae* CNCM I-3856, which can help inform the development of effective combinations of lactobacilli and yeast probiotics. Furthermore, the observed differences between strains of lactobacilli highlight the potential importance of the endogenous female microbiome in modulating the action of *S. cerevisiae* CNCM I-3856 against *C. albicans*, which can help in the development of efficient personalized choices of probiotic therapies, although this further needs to be studied in vivo.

### Supplementary Information

Supplementary Information.

## Data Availability

Original datasets generated and analyzed in this study can be made available upon request directed to the corresponding author.
