# Supplementary material for: Multifactorial inhibition of Candida albicans by combinations of lactobacilli and probiotic Saccharomyces cerevisiae CNCM I-3856
Source: Sci Rep. 2024 Apr 23;14:9365. doi: 10.1038/s41598-024-59869-9 (PMC11039719; doi:10.1038/s41598-024-59869-9)
Supplement: Supplementary file 1 — Supplementary Information. [file 41598_2024_59869_MOESM1_ESM.pdf]

Supplementary Material

Multifactorial inhibition of *Candida albicans* by combinations of lactobacilli and probiotic *Saccharomyces cerevisiae* CNCM I-3856

Authors: I. Spacova[1], C. N. Allonsius[1], I. De Boeck[1], E. Oerlemans[1], I. Tuyaerts[1], N. Van de Vliet[1], M. F. L. van den Broek[1], L. Jimenez[2], M. Boyer[2], B. Rodriguez[3], N. Ballet[2], S. Lebeer[1*]

[1]Research Group Environmental Ecology and Applied Microbiology, Department of Bioscience Engineering, University of Antwerp, Groenenborgerlaan 171, Antwerp, 2020, Belgium

[2]Lesaffre International, Lesaffre Group, Rue Gabriel Péri 137, Marcq-en-Baroeul, 59700, France

[3]Gnosis by Lesaffre, Lesaffre Group, Rue Gabriel Péri 137, Marcq-en-Baroeul, 59700, France

*** Correspondence:** Sarah Lebeer: sarah.lebeer@uantwerpen.be

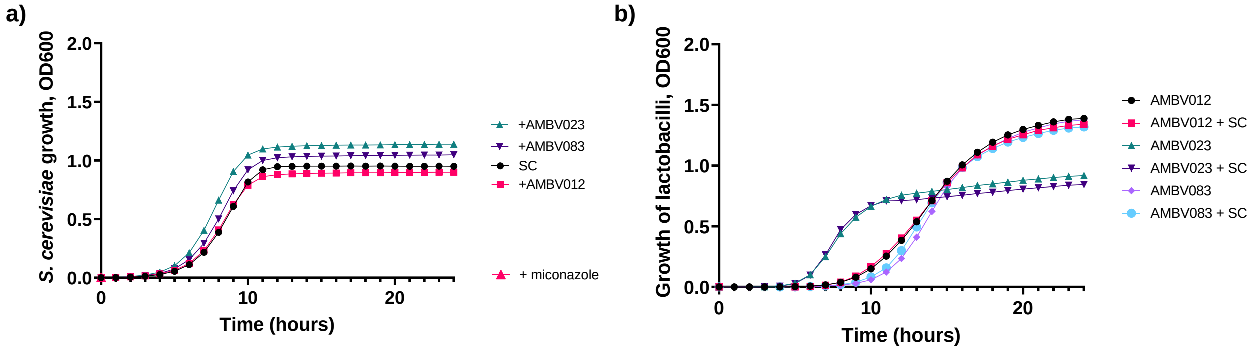

Supplementary Figure S1. Growth curves of a) *S. cerevisiae* CNCM I-3856 (SC) under the influence of the supernatant from vaginal lactobacilli isolates *Lactobacillus crispatus* AMBV012 (+AMBV012), *Lactobacillus johnsonii* AMBV023 (+AMBV023) and *Lacticaseibacillus rhamnosus* AMBV083 (+AMBV083), and b) of vaginal lactobacilli isolates under the influence of *S. cerevisiae* CNCM I-3856 supernatant (+SC conditions), obtained in the time-course growth analysis.

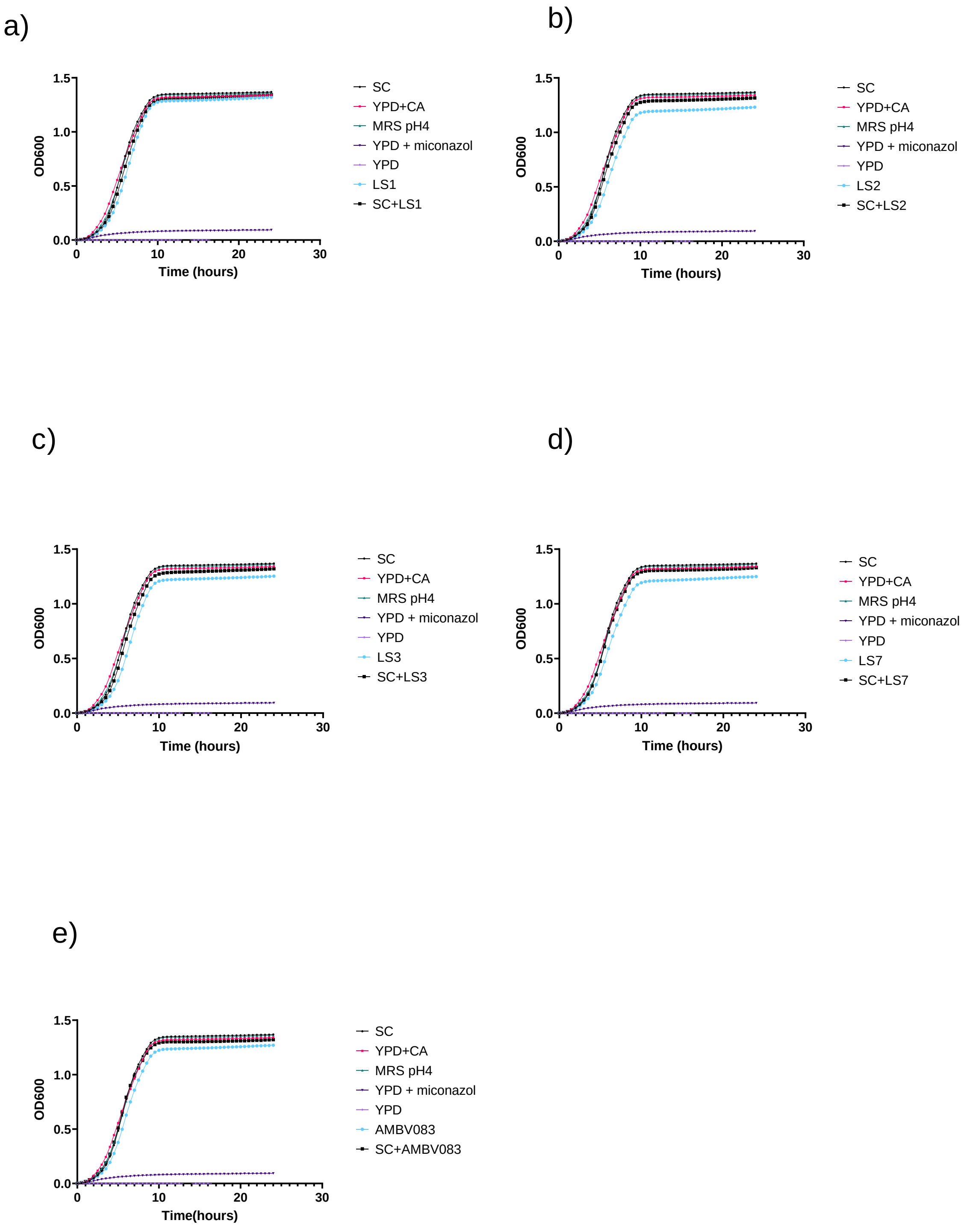

Supplementary Figure S2. Growth curves of *C. albicans* SC5314 obtained in the time-course growth assay under the influence of the supernatants of the best performing lactobacilli and the mixture of lactobacilli and *S. cerevisiae* CNCM I-3856 (SC) supernatant. The lactobacilli strains included: a) LS1, b) LS2, c) LS3, d) LS7 and e) AMBV083.

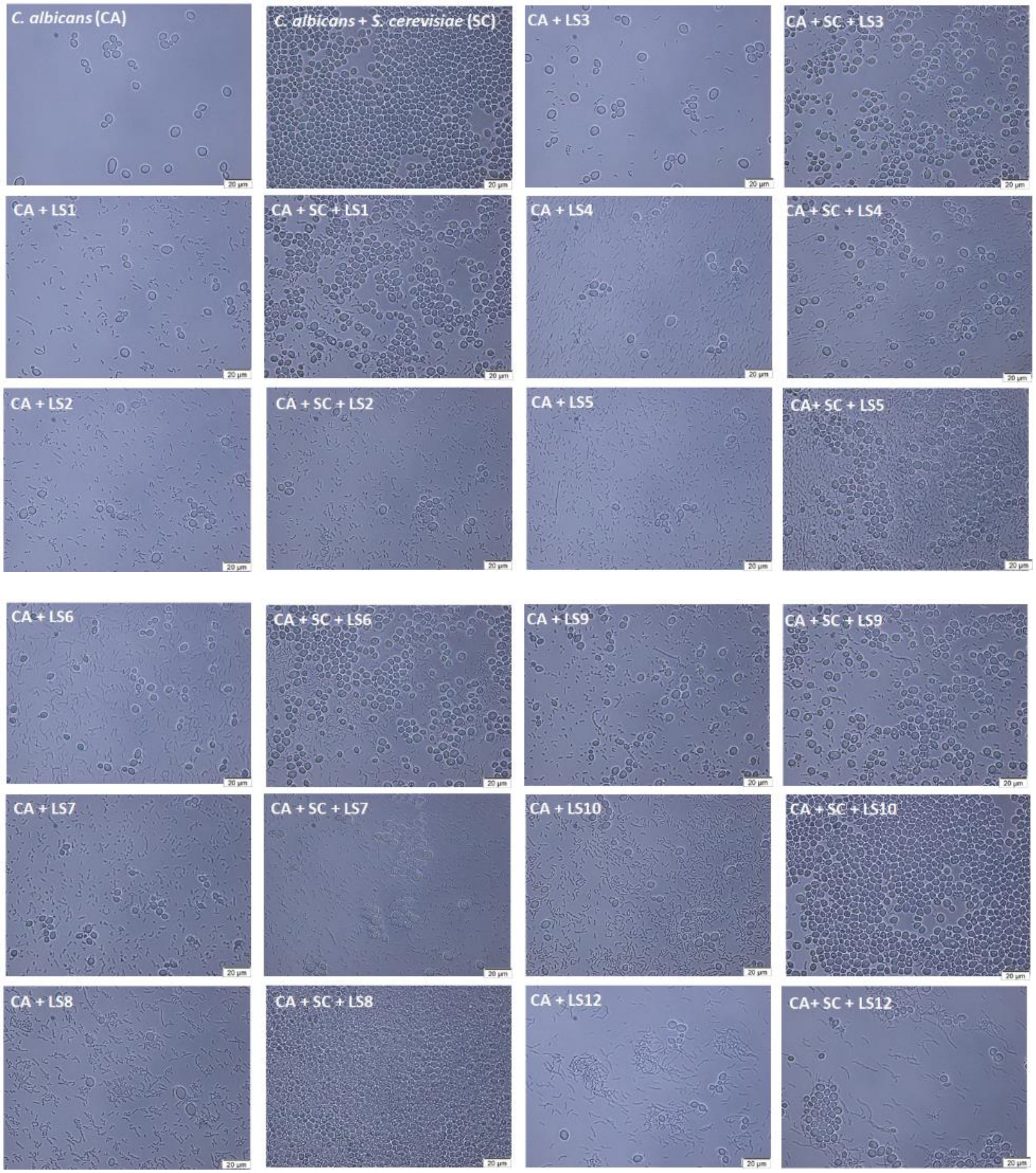
C. albicans (CA)
C. albicans + S. cerevisiae (SC)
CA + LS3
CA + SC + LS3
CA + LS1
CA + SC + LS1
CA + LS4
CA + SC + LS4
CA + LS2
CA + SC + LS2
CA + LS5
CA+ SC + LS5
CA + LS6
CA + SC + LS6
CA + LS9
CA + SC + LS9
CA + LS7
CA + SC + LS7
CA + LS10
CA + SC + LS10
CA + LS8
CA + SC + LS8
CA + LS12
CA+ SC + LS12

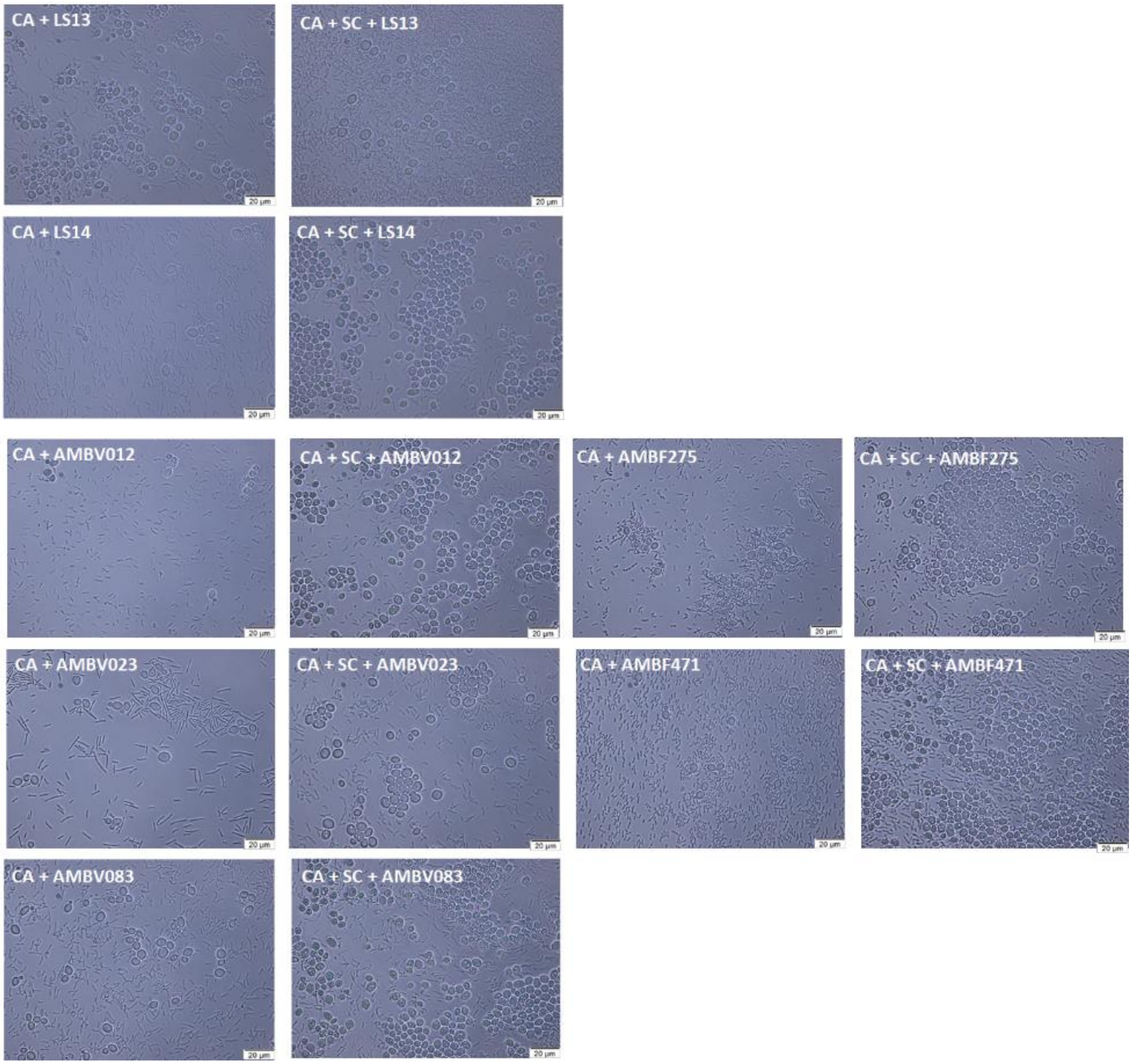

Supplementary Figure S3. Co-aggregation of *C. albicans* SC5314 cells with lactobacilli as such, or in co-culture with dried *S. cerevisiae* CNCM I-3856 after 1 hour incubation. Samples were visualized with the Olympus CX41 microscope and Olympus U-CMAD3 camera.

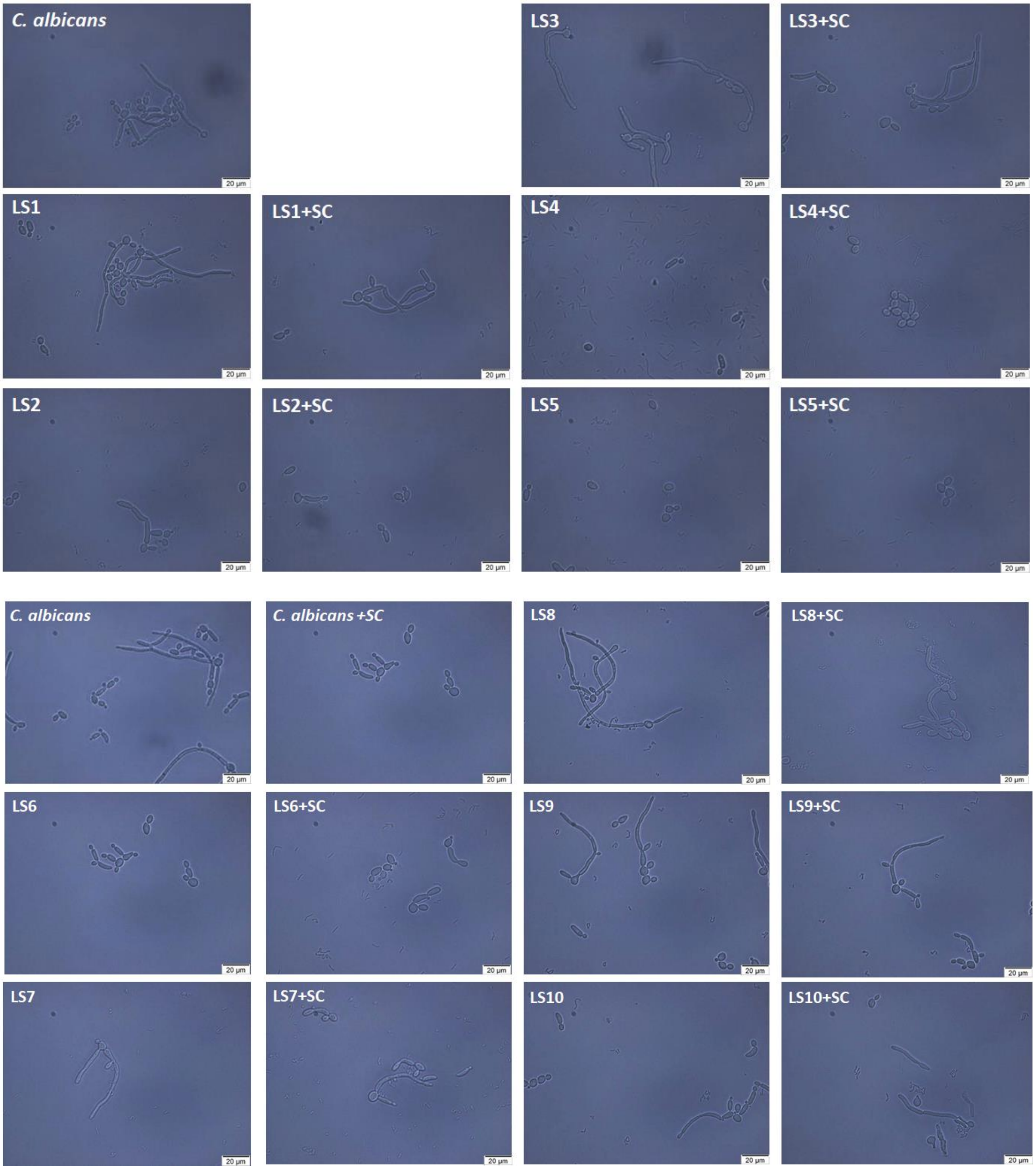

Supplementary Figure S4. Microscopic visualization of hyphal induction of *C. albicans* SC5314 during co-incubation with lactobacilli as such, or with cell-free supernatant of *S. cerevisiae* CNCM I-3856 (SC). A concentration of 1/4 FCS was used to induce hyphae. Scale bar = 20 µm. Samples were visualized with the Olympus CX41 microscope and Olympus U-CMAD3 camera. Due to the used growth medium, YPD, the contrast of the figures is less clear, as YPD has a dark color itself.

Supplementary Table S1. Aggregation scores of *C. albicans* SC5314 cells with lactobacilli as such, or in co-culture with dried *S. cerevisiae* CNCM I-3856 after 10 min of incubation. Asterisks indicate that big clusters of lactobacilli where present around the yeasts. The intensity of the green color indicates the aggregation strength (from grey (0) no aggregation to dark green (4) strongest aggregation).

10MIN	- *S. CEREVISIAE*			+ *S. CEREVISIAE*		
C. ALBICANS	0	0	0	1	1	1
**LS1**	0	0	0	2	2	2
**LS2**	1	0	0	2	2	2
**LS3**	1	1	1	2	2	2
**LS4**	1	1	1	2	2	2
**LS5**	0	0	0	2	2	1
**LS6**	1	1	0	2	2	2
**LS7**	0	0	0	2	2	2
**LS8**	0	0	0	2	2	2
**LS9**	1	1	0	2	2	2
**LS10**	0	0	0	2	2	2
C. ALBICANS	0	0	0	1	1	1
**LS11**	/	/	/	/	/	/
**LS12**	1	1	1	2*	2*	2*
**LS13**	1	1	1	1	1	1
**LS14**	1	1	1	1*	2*	2*
**AMBV012**	1	1	1	2	2	2
**AMBV023**	1	1	1	2	2	2
**AMBV083**	1	1	1	2	2	2
**AMBF275**	2*	2*	2*	2	2	2
**AMBF471**	1	1	1	2	2	2

Supplementary Table S2. Aggregation scores of *C. albicans* SC5314 cells with lactobacilli as such, or in co-culture with dried *S. cerevisiae* CNCM I-3856 after 1 hour of incubation. Asterisks indicate that big clusters of lactobacilli where present around the yeasts. The intensity of the green color indicates the aggregation strength (from grey (0) no aggregation to dark green (4) strongest aggregation).

1 HOUR	- *S. CEREVISIAE*			+ *S. CEREVISIAE*		
C. ALBICANS	1	1	1	4	4	4
**LS1**	1	1	1	2	3	3
**LS2**	1	1	1	2	2	2
**LS3**	1	1	1	3	3	4
**LS4**	1	1	1	2	2	2
**LS5**	1	1	1	3	2	3
**LS6**	1	1	1	3	2	4
**LS7**	0	1	1	2	2	2
**LS8**	0	0	0	2	3	4+
**LS9**	1	0	1	2	3	2
**LS10**	2	1	1	4	4	4
C. ALBICANS	1	1	1	3	3	3
**LS11**	/	/	/	/	/	/
**LS12**	1	1	1	3*	3*	2*
**LS13**	1	1	1	2*	2*	3*
**LS14**	1	1	1	3*	3*	3*
**AMBV012**	1	1	1	2	3	2
**AMBV023**	1	1	1	2	2	3
**AMBV083**	1	2*	1	2	3	2
**AMBF275**	2*	2*	2*	3	3	3
**AMBF471**	2	1	1	2	3	3
